# Ubiquitin and Not Only Unfolded Domains Drives Toscana Virus Non-Structural NSs Protein Degradation

**DOI:** 10.3390/v12101153

**Published:** 2020-10-12

**Authors:** Gianni Gori Savellini, Luca Bini, Assunta Gagliardi, Gabriele Anichini, Claudia Gandolfo, Shibily Prathyumnan, Maria Grazia Cusi

**Affiliations:** 1 Department of Medical Biotechnologies, University of Siena, 53100 Siena, Italy; gabriele.anichini@student.unisi.it (G.A.); claudia.gandolfo@unisi.it (C.G.); shibilyps@gmail.com (S.P.); mariagrazia.cusi@unisi.it (M.G.C.); 2 Department of Life Sciences, University of Siena, 53100 Siena, Italy; luca.bini@unisi.it; 3 Department of Cellular, Computational and Integrative Biology (CIBIO), University of Trento, Laboratory of Synthetic and Structural Vaccinology, 38122 Trento, Italy; assunta.gagliardi@unitn.it; 4 S. Maria delle Scotte Hospital, V.le Bracci, 1, 53100 Siena, Italy

**Keywords:** ubiquitin-proteasome system, NSs protein, protein stability

## Abstract

The non-structural protein NSs of the *Phenuiviridae* family members appears to have a role in the host immunity escape. The stability of Toscana virus (TOSV) NSs protein was tested by a cycloheximide (CHX) chase approach on cells transfected with NSs deleted versions fused to a reporter gene. The presence of intrinsically disordered regions (IDRs) both at the C- and N-terminus appeared to affect the protein stability. Indeed, the NSsΔC and NSsΔN proteins were more stable than the wild-type NSs counterpart. Since TOSV NSs exerts its inhibitory function by triggering RIG-I for proteasomal degradation, the interaction of the ubiquitin system and TOSV NSs was further examined. Chase experiments with CHX and the proteasome inhibitor MG-132 demonstrated the involvement of the ubiquitin-proteasome system in controlling NSs protein amount expressed in the cells. The analysis of TOSV NSs by mass spectrometry allowed the direct identification of K_104_, K_109_, K_154_, K_180_, K_244_, K_294_, and K_298_ residues targeted for ubiquitination. Analysis of NSs K-mutants confirmed the presence and the important role of lysine residues located in the central and the C-terminal parts of the protein in controlling the NSs cellular level. Therefore, we directly demonstrated a new cellular pathway involved in controlling TOSV NSs fate and activity, and this opens the way to new investigations among more pathogenic viruses of the *Phenuiviridae* family.

## 1. Introduction

Toscana virus (TOSV) is a member of the *Phenuiviridae* family (*Phlebovirus genus*) classified as an emergent sandfly-borne virus. It is mainly transmitted to humans by *Phlebotomus perfiliewi*, *P. perniciosus*, and *P. papatasi* sandfly species [1,2,3]. Although pauci-symptomatic infections are described in endemic countries [4], TOSV infection is mostly associated to meningitis or more severe central nervous system (CNS) injuries, such as encephalitis and cerebral ischemia [4,5,6]. Nowadays, TOSV is widely present in the Mediterranean basin [7,8,9,10,11] and represents a significant public health threat.

The non-structural protein (NSs) of the *Phenuiviridae* and *Bunyaviridae* family members represents an important virulence factor, inhibiting the host innate immunity to viral infections, mainly mediated by type I interferons (IFN-α/β) [12,13,14,15,16,17,18,19,20,21]. In order to overcome this first-line defense implemented by the host, viruses evolved protein(s) able to block the IFN-β production and its downstream activity at different steps in the signaling cascade.

However, TOSV is the first Phlebovirus described to date, whose behavior is different from that observed among the *Bunyaviridae* or *Phenuiviridae* members, since interferons are not inhibited during viral infection and replication, despite its NSs protein. TOSV NSs protein is rapidly degraded by the ubiquitin-proteasome system, as previously demonstrated [19,20,21]. Therefore, during TOSV infection in humans, the ubiquitination and degradation of the NSs protein occur very early in virus replication to prevent IFN-β inhibition in the host.

The proteasomal degradation of proteins is triggered by ubiquitination, a process consisting of covalent attachment of poly-ubiquitin (poly-Ub) chains at lysine residues on the target protein. The assembly of poly-Ub chains to the target protein is accomplished by the cooperation of ubiquitin-activating enzymes (E1), ubiquitin-conjugating enzymes (E2), and ubiquitin-ligases (E3), which work in a sequential cascade [22,23,24,25,26,27,28,29,30,31,32,33,34]. A well-characterized cellular complex, which mediates ubiquitination of target proteins, is represented by the Skp, Cullin, and F-box (SCF)-containing complex. Cullin activity is regulated by their NEDdylation, which is the covalent attachment of the small ubiquitin-like protein NEDD8 (neural precursor cell expressed developmentally downregulated 8) to the cullin subunit via the NEDD8 activating enzyme (NAE) [25,26]. In this context, the E3 ubiquitin ligase is the only enzyme that confers specificity to this system by recognizing selected target proteins [24,25,26].

The structure of the poly-Ub chain assembled by the E3 ligase is crucial for target protein fate and function [22,33]. Covalent bonding between ubiquitin monomers occurs at one of the seven lysine residues in the previously attached ubiquitin molecule, resulting in the formation of ubiquitin chains containing distinctive linkages between the ubiquitin moieties, thus creating a different structure. Based on the linkage generated between ubiquitin moieties, the cognate proteins undergo regulation of their physiological functions, although the role of some chains is still elusive [34,35,36,37,38]. Notably, Lys_48_ (K_48_) ubiquitin linkage has been reported to be involved in targeting proteins for degradation by the 26S proteasome, while the Lys_63_ (K_63_) linkage has been proved to regulate protein functions, especially those involved in signal transduction, cell cycle, and gene expression [23,28,31]. So far, the involvement of the ubiquitin system in virus replication, latency, oncogenic properties, and immunity escape has been widely demonstrated [39,40,41,42,43,44,45,46,47,48,49,50,51,52,53,54,55,56,57,58,59].

Among *Phenuiviridae* members, Rift Valley fever virus (RVFV) is the most investigated virus in terms of antagonistic effects of its NSs protein. The involvement of the ubiquitin system, and in particular of the SCF E3 ubiquitin ligase complex, has been recently elucidated [59,60,61]. However, despite the involvement of RVFV NSs in the ubiquitin-proteasome control of cellular components, no direct evidence of its ubiquitination and fate/function regulation has been shown.

Regarding TOSV, the involvement of the ubiquitin system in controlling its NSs activity was further demonstrated by a recent work, where an E3 ubiquitin ligase activity has been attributed to the viral protein. Similarly to RVFV, this E3 ligase activity was necessary to mediate RIG-I ubiquitination and proteasomal degradation and, consequently, impede IFN-β production [57]. The only evidence that Bunyaviridae NSs protein could be subjected to ubiquitination has been investigated in the Bunyamwera virus [62,63]. Indeed, analysis of recombinant virus carrying lysine knockdown NSs variant highlighted the increased stability of the mutated protein.

However, no significant advantage in virus growth and virulence in mice were reported, suggesting that NSs ubiquitination is not essential for the virus life cycle [62].

Here, we reported the first evidence of TOSV NSs ubiquitination. Mass spectrometry analysis allowed the identification of lysine residues 104, 109, 154, 180, 244, 294, and 298 on the NSs targeted for ubiquitination. The influence of these sites on protein stability was deeply investigated to evidence their role in protein function and stability, along with the effects of disordered regions located at the C- and N-terminus of the protein.

## 2. Materials and Methods

### 2.1. Cells and Viruses

Human embryonic kidney Lenti-X 293T cells (Clontech, Milan, Italy) were cultured in Dulbecco’s modified Eagle’s medium (DMEM) (Lonza, Milan, Italy) supplemented with 100 U/mL penicillin/streptomycin (Hyclone Europe, Milan, Italy) and 10% heat-inactivated fetal calf serum (FCS) (Lonza), at 37 °C. Toscana virus (TOSV) strain 1812 [64] was used as a template source for NSs cloning described where the N-terminal deleted (NSsΔN) NSs protein variant was generated.

### 2.2. Reagents and Antibodies

Transient transfections were performed with GeneJuice^®^ Transfection reagent (Novagen, Milan, Italy), according to the manufacturer’s instructions, or standard calcium phosphate method. The proteasome inhibitor MG-132 and cycloheximide (CHX) were purchased from Sigma-Aldrich (Milan, Italy). Mouse anti-6×His tag antibody (GE Healthcare, Milan, Italy), mouse monoclonal anti-HA tag antibody, and HRP-conjugated anti-mouse IgG were purchased from Sigma-Aldrich. Ni-NTA sepharose was from Novagen (Milan, Italy).

### 2.3. Plasmids

Six His-tagged full-length TOSV NSs expression vector was described elsewhere [58]. Amino-terminal-deleted (NSsΔN) and carboxy-terminal-deleted (NSsΔC) NSs protein variants were generated by cloning the nt: 217–948 and nt: 1–537 sequences on TOSV 1812 strain (GenBank: EU327772.1) in-frame into the pcDNA4HisMax (Life Technologies, Milan, Italy) expression plasmid. Having arginine instead of lysine 104, 108, 109, 150, 154, and 179, NSs mutant (NSs-6KR) was generated by using QuikChange II Site-Directed Mutagenesis Kit (Agilent Technologies, Milan, Italy) according to the manufacturer’s instructions. NSs variants with lysine residues at the N-terminus (9, 17, 36, 57, 59, and 69) mutated to arginine (NSs-NKR and NSs-6KR-NKR) were generated substituting the N-terminal part with a mutated synthetic fragment (gBlocks, IDT Integrated DNA technologies, Leuven, Belgium). To measure NSs mutant proteins’ stability, FireFly Luciferase (FFLuc) fused proteins were generated. FFLuc-NSs and FFLuc-NSsΔC were already described elsewhere [58]. A similar approach was used to obtain the FFLuc-NSs new variants. Briefly, the NSs recombinant plasmids were linearized with BamHI and the FFLuc coding gene was inserted upstream and in frame with the NSs gene by the InFusion system (Clontech, Milan, Italy) following the manufacturer’s instructions. All the recombinant plasmids were verified by sequencing. The *Renilla* Luciferase reporter plasmid (pSV40-RL) was purchased by Promega (Promega, Milan, Italy). HA-tagged ubiquitin expressing plasmid was a kind gift of D. Arnoult (Inserm, France) while K48-only and K63-only ubiquitin plasmids were purchased from Addgene (Teddington, UK).

### 2.4. Cycloheximide Chase Analysis and NSs Protein Stability

Lenti-X 293T cells were seeded in 6-well plates and, after 24 h, transfected with the FFLuc-fused wt-; deleted or mutated NSs expressing plasmids along with 200 ng of pRL-SV40 (Promega) for normalization. Twenty-four hours later, transfected cells were split in a 24-well plate, in triplicate and, after additional 12 h, cells were treated with 25 μM MG-132 for 30 min or untreated. After the inhibitor treatment, in order to start protein expression quantification (T0), cell samples were collected, while the remaining samples were exposed to 100 μg/mL of CHX, or 25 μM MG-132 or 25 μM MG-132 along with 100 μg/mL of CHX. Samples were collected 1.5 and 3 h later for time course quantification. FFLuc-fusion protein amount, detected after translation inhibition by CHX, was quantified by measuring FFLuc activities. Lysates and assay set-up were prepared according to Dual-Luciferase reporter assay (Promega). Relative FFLuc values were normalized with respect to the corresponding RL activities (FFLuc/RL), then fold changes of each sample were calculated with regard to the corresponding T0, mock-treated, sample. The deduced half-life of each protein was calculated with tools available on the web (https://www.calculator.net/half-life-calculator.html).

### 2.5. Pull-Down and Immunoblot Analysis

Lenti-X 293T cells, seeded in T25 flasks, were transfected with 4 µg of NSs expressing plasmid and, where indicated, with HA-tagged ubiquitin mutants by using standard calcium phosphate precipitation protocol [65]. Thirty-six hours later, cells were treated with 1 μM MG-132 for additional 12 h and collected 48 h post-transfection. Enrichment of the NSs protein was achieved by Immobilized Metal Affinity Chromatography (IMAC) under denaturing conditions [66]. Briefly, cell pellets were lysed in 5 M guanidine-HCl; 10 mM HEPES (pH 8.0) with sonication to share genomic DNA. His-tagged NSs was bound to Ni-NTA sepharose overnight (o/n) at 4 °C. Beads were collected and washed three times with 10 volumes of 10 mM HEPES (pH 8.0), 1 M NaCl, 0.3% w/v N-lauroylsarcosine (SRK), 20 mM imidazole. Bound proteins were eluted by washing buffer supplemented with 500 mM imidazole. An aliquot of eluted sample was loaded on SDS-PAGE, transferred to nitrocellulose membrane (Santa Cruz Biotechnology, Heidelberg, Germany) and processed for immunoblotting. Briefly, membrane blocking was accomplished with 5% non-fat dry milk, then filters were incubated o/n at 4 °C with anti-NSs (1:200 dilution) or mouse anti-HA monoclonal antibody (1:1000 dilution) (Sigma-Aldrich). After being washed with PBS 0.2% Tween-20 (PBS-T), membranes were incubated with a selected secondary antibody (1:5000 dilution). Immunocomplexes were detected with TMB Enhanced One Component HRP Membrane Substrate (Tebu-bio, Milan, Italy). Ubiquitin modification of the NSs was evidenced by a shift in its MW (10 kDa for mono-ubiquitination, 20 kDa for di-ubiquitination, 10.5 multiples for poly-ubiquitination).

### 2.6. Mass Spectrometry Detection of NSs Ubiquitination

NSs protein was enriched from Lenti-X 293T-transfected cells. Proteins were separated by SDS-PAGE and stained with Bio-safe Coomassie stain (Bio-Rad, Milan, Italy). Protein bands corresponding to potentially ubiquitinated NSs isoforms were manually cut from gel and prepared for mass-spectrometry analysis. Each band was first destained with 2.5 mM ammonium bicarbonate and 50% acetonitrile, and dehydrated in 100% acetonitrile. A reduction and alkylation procedure was then applied, using 10 mM DTE in 25 mM ammonium bicarbonate for 1 h at 56 °C, followed by incubation in 55 mM iodoacetamide and 25 mM ammonium bicarbonate at room temperature for 45 mins, in dark room. Protein bands were rinsed for 10 mins with 50 mM ammonium bicarbonate and dehydrated, again, with 100% acetonitrile. Dried gels were rehydrated in trypsin solution (Sigma Aldrich, Italy) and in-gel protein digestion was performed overnight at 37 °C. Protein identification was carried out by Peptide Mass Fingerprinting (PMF) on an ultrafleXtreme™ MALDI-TOF/TOF mass spectrometer (Brucker Corporation, Billerica, MA, USA). In total, 0.75 µL of each digested protein supernatant were spotted onto the MALDI target and allowed to dry. Then, 0.75 μL of matrix solution (5 mg/mL alpha-ciano 4-hydroxycynnamic acid in 50% v/v acetonitrile and 0.5% v/v trifluoroacetic acid) were added to the dried sample and air-dried again. A PMF search was performed in NCBInr databases using MASCOT search engine available on-line (Matrix Science Ltd., London, UK, http://www.matrixscience.com). The following parameters were used: taxonomy was limited to viruses, mass tolerance was 100 ppm, the acceptable number of missed cleavage sites was set to two, alkylation of cysteine by carbamidomethylation was assumed as a fixed modification, and oxidation of methionine was considered as a possible modification. The criteria used to accept identifications included the extent of sequence coverage (>15%), the number of matched peptides (>4), and the MASCOT algorithm assigned probabilistic score (>60 or *p* < 0.001). Confirmatory results were also obtained by analysis of the same samples, carried out by Cogentech Proteomics/MS (Cogentech S.c.a.r.l., Milan, Italy) and the mass spectrometry facility at the Toscana Life Sciences (TLS, Siena, Italy) by using the nLC-ESI-MS/MS QExactive-HF system.

## 3. Results

### 3.1. NSs Stability Is Influenced by Disordered Regions

**Figure 1:**
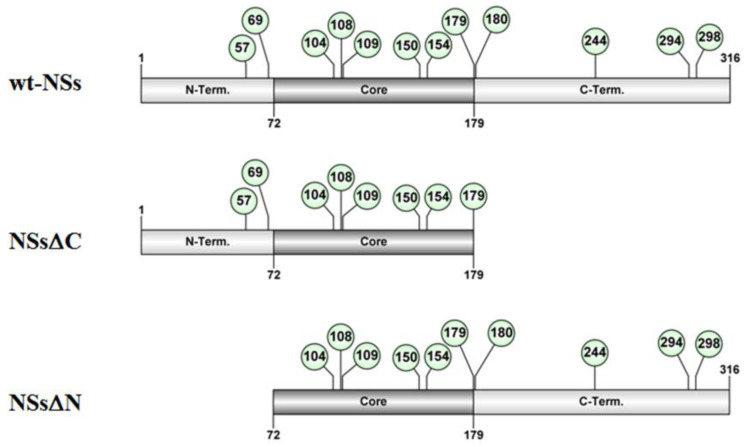
Schematic representation of the of TOSV NSs full-length (wt-NSs) sequence, N-(NSsΔN), or C-terminus deleted (NSsΔC) variants. Green dots indicate the lysine residues with a high predictive score for ubiquitination.

Putative intrinsically disordered regions (IDRs) were identified in TOSV NSs by on-line tools (http://prdos.hgc.jp). Based on a predictive algorithm, two IDRs were mapped at aa 1–17 of the N-terminus and aa 295–316 of the C-terminus of the protein. Previous results already showed the important role of the C-terminus, since its deletion influenced protein stability [58]. Next, we assessed the role of the N-terminus on the NSs protein stability by deleting the first 72 aa (Figure 1).

**Figure 2:**
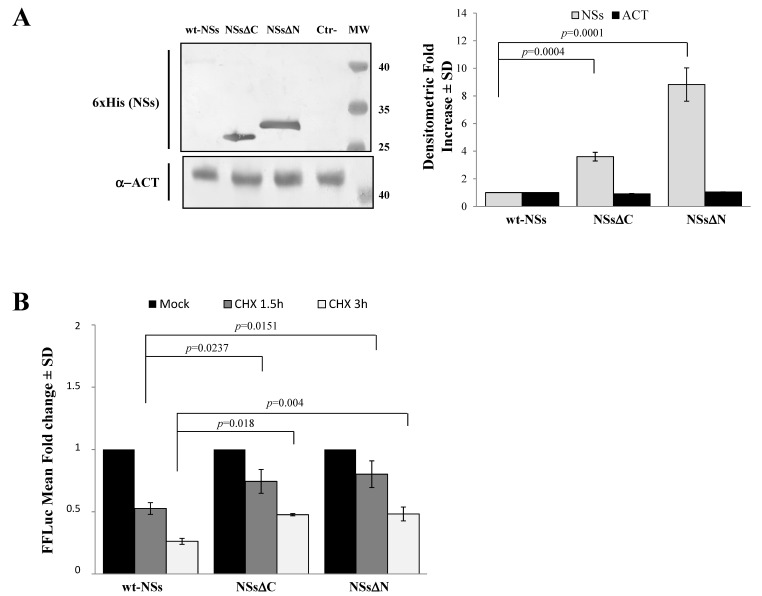
Domains affecting TOSV NSs stability. (**A**) The involvement of TOSV NSs C- and N-terminal regions on protein stability was demonstrated by generating deleted NSs proteins (NSsΔC and NSsΔN). The behavior of the deleted NSs variants was tested by immunoblotting on the whole-cell lysates (50 μg) of Lenti-X 293T-transfected cells. Specific proteins were detected by using anti-6×His (NSs) monoclonal antibody (left figure). Loading control was represented by actin (α-ACT) detection (left figure). Quantitative assessment of deleted NSs variants was determined by densitometric analysis (right figure). (**B**) Lenti-X 293T cells were transfected with FFLuc NSsΔN and NSsΔC fusion constructs and *Renilla* Luciferase as an internal control. Transfected cells were mock-treated or treated with cycloheximide (CHX) and collected at 1.5 and 3 h. Fold induction was obtained by luciferase activity normalization with respect to *Renilla* luciferase values and comparison to the relative mock-treated sample. Results were expressed as mean fold change values collected in at least three independent experiments ± standard deviation.

Immunoblotting and densitometric analysis of lysates of Lenti-X 293T cells transfected with NSs expressing plasmid showed a 9-fold increase of NSsΔN protein accumulation compared to the wt-NSs protein (*p* ≤ 0.0005) (Figure 2A), confirming the presence of a disordered instable region at the N-terminus.

To better address the involvement of N-terminus IDR on the NSs stability, Firefly Luciferase (FFLuc) fusion proteins were generated. Afterwards, cycloheximide (CHX) chase experiments were performed to compare protein stability among the NSsΔN, NSsΔC, and wt-NSs. Luciferase activities were measured in transfected CHX-treated cells. After normalization with respect to the constitutively expressed *Renilla* luciferase (pSV40-RenLuc), a considerable reduction of the Luciferase activities, consistent with NSs degradation, was reported in wt-NSs lysates just 1.5 h after CHX treatment in comparison with the mock-treated sample. On the contrary, the detection of a higher Luciferase signal for NSsΔN and NSsΔC demonstrated a significant increased protein stability at both 1.5 and 3 h after CHX treatment (Figure 2B). Moreover, based on the CHX chase experiment datasets, the deduced half-lives of NSsΔN (t_1/2_ 8.7 h) and NSsΔC (t_1/2_ 4.8 h) were significantly longer (*p* < 0.0001) than those observed for wt-NSs (t_1/2_ 1.6 h) (Data not shown). These data support the prediction of intrinsic disordered sequences located at the terminal ends of the NSs, thus the deleted variants of the protein acquired greater stability and cytoplasmic accumulation in transfected cells.

### 3.2. Ubiquitin-Dependent NSs Proteasomal Degradation

**Figure 3:**
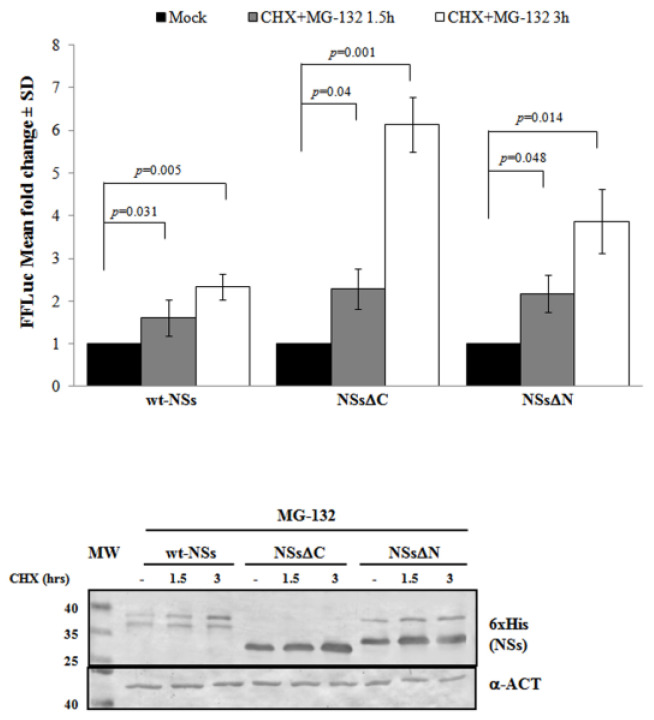
Effects of the proteasome inhibitor MG-132 on NSs deleted mutants were evaluated. (**Upper panel**) wt-NSs, NSsΔN, and NSsΔC expressing cells were treated with 25 μM of the inhibitors along with 100 μg/mL of CHX and collected at 1.5 and 3 h. Cell lysates were subjected to a dual-luciferase assay in order to estimate the stability of NSs protein variants. A significant increase of protein stability over time was noticed for wt-NSs, NSsΔC, and NSsΔN. (**Lower panel**) The immunoblotting with anti-6×His antibody or anti-ACT performed on MG-132 and CHX treated cells confirmed the stabilizing properties of the MG-132 on the NSs proteins tested. The error bars represent the standard deviation from the mean values obtained in independent experiments.

Previous results have shown that TOSV NSs retains antagonistic function on host innate immunity to viral infection [20,21,58] exhibiting an E3 ubiquitin ligase activity on RIG-I [57]. Therefore, we also investigated the effect of ubiquitination on the fate and function of the viral protein. Similarly to Bunyamwera virus, TOSV NSs protein stability was also evaluated analyzing its possible ubiquitination, since its accumulation into the cell cytoplasm was strongly enhanced by the exposure to the proteasome inhibitor MG-132 [20,21]. Indeed, a significant increase of protein stability (*p* < 0.05) was noticed when the inhibitor MG-132 was included during the CHX chase experiments, with a fold increase of protein accumulation at 3 h treatment of 2.3 for the wt-NSs, 6.1 for NSsΔC, and 3.9 for NSsΔN (Figure 3). Moreover, the immunoblotting confirmed the enhanced protein accumulation in the cell cytoplasm when the transfected cells were exposed only to MG-132 (Figure 3).

Furthermore, the positive effects of the proteasome inhibitor were evidenced by the deduced half-life of NSsΔC (t_1/2_ 22 h) and NSsΔN (t_1/2_ 26.4 h), which was significantly higher (*p* < 0.05) with respect to the untreated counterparts. This evidence confirmed the ubiquitinated status of TOSV NSs, suggesting that the stability of TOSV NSs was also controlled by ubiquitination and proteasomal degradation and that lysine residues target for ubiquitination were at least located in the central region of the protein, common to the three constructs.

### 3.3. Evidence of TOSV NSs Ubiquitination

**Figure 4:**
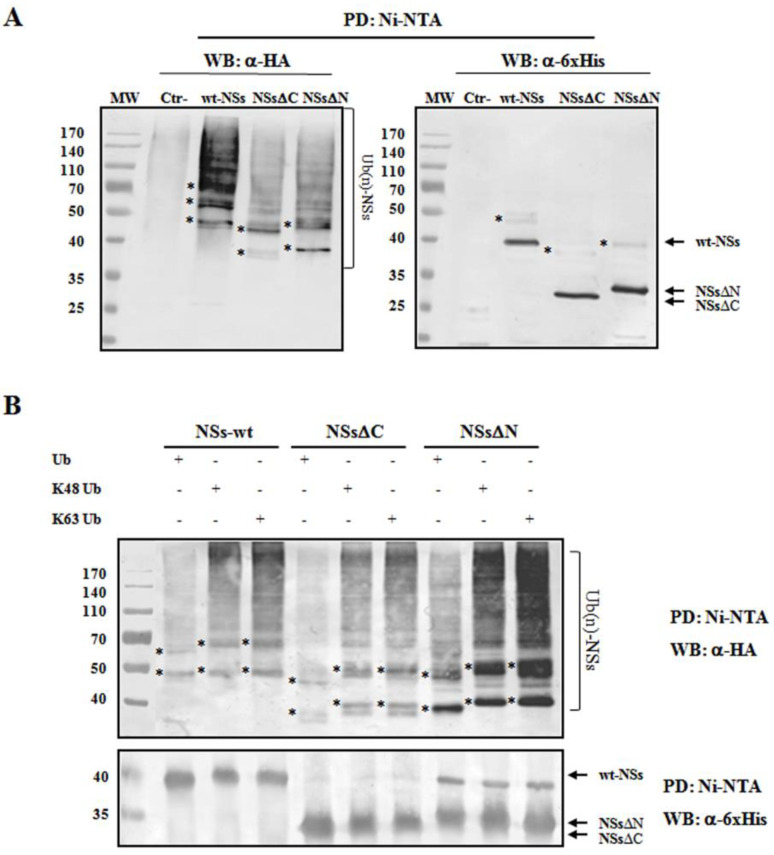
TOSV NSs undergoes ubiquitination. NSs ubiquitination was evaluated by immunoblotting. (**A**) Lenti-X 293T cells were transfected with wt-, ΔC-, or ΔN-expressing plasmids, along with the plasmid expressing HA-tagged wild-type ubiquitin (Ub). Cells treated with the proteasome inhibitor MG-132 were collected at 48 h post-transfection and NSs protein enrichment was performed on cell lysates by pull-down (PD) experiments using Ni-NTA agarose beads. 6×His-NSs-enriched samples were subjected to immunoblotting for Ub (α-HA) or NSs (α-6×His) detection. The ubiquitinated status of the three NSs forms was evaluated as a modification of the targeted substrate, causing a shift in MW of ~10 kDa (mono-ubiquitination) or multiples. Asterisk represents ubiquitinated NSs forms. (**B**) The rate on K_48_-and K_63_-moiety ubiquitination was assessed by PD assay and immunoblotting. Lenti-X 293T cells were transfected with wt-, ΔC, or ΔN NSs expressing plasmids, along with the plasmid expressing HA-tagged K_48_-only or K_63_-only ubiquitin mutants. Twenty-four hours later, cells were treated with MG-132 and collected after additional 24 h. Lysates were prepared and PD with Ni-NTA agarose beads. Isolated proteins were separated by SDS-PAGE and probed by immunoblotting for NSs (α-6×His) and Ub-K_48_ and Ub-K_63_ (α-HA) detection. Asterisk indicates ubiquitinated forms of the NSs proteins.

To understand whether TOSV NSs was directly ubiquitinated, the presence of polyubiquitin chains linked to the viral protein was investigated. The denaturant pull-down assay performed on NSs and HA-Ub co-transfected cells allowed the efficient inactivation of de-ubiquitinating enzymes (DUBs), preserving NSs ubiquitinated forms [60]. The ubiquitination status of the affinity purified NSs was detected by immunoblotting using anti-6×His and anti-HA antibodies, demonstrating that NSs protein underwent a robust ubiquitination. Indeed, high-molecular-weight migrating bands with a constant increase were detected with anti-HA monoclonal antibody, corresponding to mono-, multi-, or poly-ubiquitinated forms of NSs (Figure 4A). Unfortunately, the anti-6×His monoclonal weakly detected these bands due to a lower sensitivity of the antibody.

As shown in Figure 4A, both the N- and C-terminal-truncated proteins underwent ubiquitination at a similar extent to that observed for the wt-NSs. On the basis on these results, it appears that lysine residues target for ubiquitination are located in the central region of the protein. We further investigated the ubiquitin-linkage type present on the NSs protein, particularly the K_48_- or K_63_-chain. These experiments demonstrated that both K_48_- and K_63_-ubiquitination moiety occurs in both wt- and the deleted NSs variants (Figure 4B). Indeed, anti-HA reactive bands corresponding to mono-, multi-, or poly-ubiquitinated forms of the NSs were detected in all the samples tested. These data supported the idea that both K_63_ and K_48_ ubiquitin linkages take place, thus this type of post-translational modification does not only influence NSs stability, but it could also affect NSs protein activity.

### 3.4. Specific NSs Lysine Residues Undergo Ubiquitination

**Figure 5:**
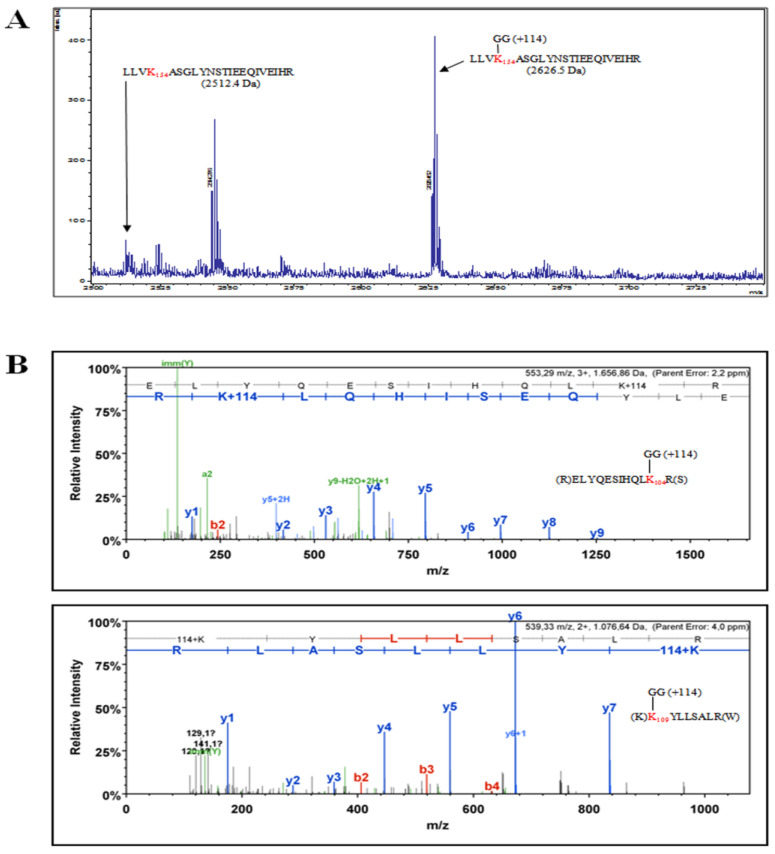
The NSs protein, enriched from cell lysate of transfected cells, was analyzed by mass spectrometry in an attempt to identify lysine residues targeted for specific ubiquitination. (**A**) MS1 mass spectrum (Department of Life Sciences, University of Siena, Siena) showing the identification of TOSV NSs peptide containing the ubiquitinated lysine residue at position 154 and (**B**) MS2 mass spectrum (Cogentech S.c.a.r.l., Milan, Italy) showing TOSV NSs peptides ubiquitinated on lysines at positions 109 and 104.

The identification of specific NSs lysine residues targeted for ubiquitination was mapped by mass spectrometry analysis. NSs protein was enriched from transfected Lenti-X 293T cells under denaturing conditions. The recovered protein was resolved by SDS-PAGE and Coomassie staining. The gel portion of interest was processed for mass spectrometry analysis. NSs peptides generated by trypsin digestion were subjected to mass spectrometry analysis. Some lysines carrying the Gly-Gly signature di-peptide were detected and assigned as being bound to ubiquitin [67]. Three lysine residues at position 104, 109, and 154, located in the central part of the NSs, were recognized as a target for ubiquitin on the recovered protein (Figure 5).

**Figure 6:**
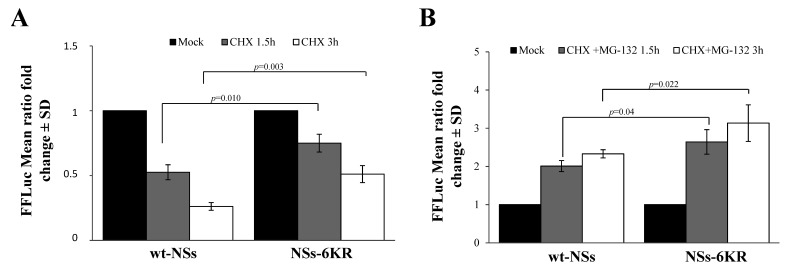
Effects of lysine residues in the core region on protein stability. (**A**) A new NSs mutant (NSs-6KR), consisting of arginine substitution at lysine 104, 108, 109, 150, 154, and 179 position, was generated and tested for protein stability. CHX chase experiments evidenced a significantly increased stability for the NSs mutant with respect to the wt-NSs protein. (**B**) The NSs-6KR mutant still responded to MG-132, as shown by the chase experiments. Transfected cells were treated with CHX in combination with 25 μM of MG-132 and then residual luciferase activities were measured. Graphs represent mean values ± standard deviations (SD) of three independent experiments.

However, we could not exclude that other lysine residues subjected to ubiquitination were located in other regions of the NSs protein. Therefore, we expressed and tested the NSs-6KR protein variant, consisting of a full-length NSs protein with mutated lysine in the core region (K_104_; K_108_; K_109_; K_150_; K_154_; and K_179_), for the protein degradation rate. As evidenced by CHX chase, the 6KR mutant protein was highly stable compared to wt-NSs (*p* = 0.003), suggesting that lysine residues targeted for ubiquitination were located in the core region of the protein (Figure 6A).

Moreover, the estimated half-life of the NSs-6KR was double (*p* = 0.0069) than that of wt-NSs (t_1/2_ 3.7h vs. t_1/2_ 1.6 h). Notwithstanding, MG-132 still affected NSs-6KR protein degradation by increasing its accumulation into the cells, leading to the conclusion that lysine residues other than those in the core region could undergo ubiquitination (Figure 6B).

**Figure 7:**
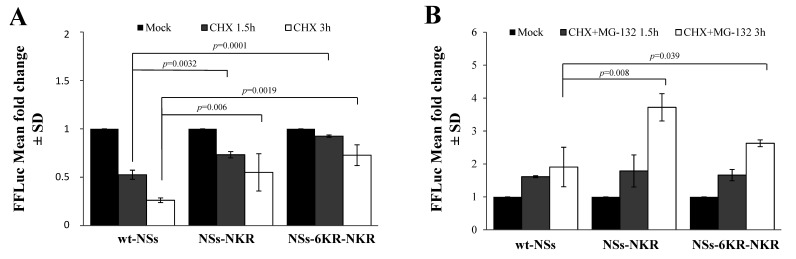
Effects of amino-terminal lysine residues on protein stability. (**A**) Lysates of NSs-NKR and NSs-6KR-NKR-transfected cells were collected at different time points after CHX exposure and subjected to the dual-luciferase assay in order to estimate the stability of NSs protein variants by measuring the FFLuc residual activities. (**B**) Cells expressing new NSs mutants, treated with 25 μM of MG-132, along with 100 μg/mL of CHX, evidenced the activity of the inhibitor on both NSs-NKR and NSs-6KR-NKR. Cells were collected at 1.5 and 3 h post-treatment and lysates were subjected to a dual-luciferase assay in order to estimate the stability of NSs protein variants. The error bars represent the standard deviation from the mean values obtained in independent experiments ± standard deviations (SD).

We then evaluated the presence of specific lysines, which might undergo ubiquitination in the N- or C-terminal part of the NSs protein. Two NSs variants were generated, mutating the lysine residues 9, 17, 36, 57, 59, and 69 at the amino-terminus of the wt protein (NSs-NKR) or of the NSs-6KR mutant (NSs-6KR-NKR). The CHX chase assessed to determine variations in protein stability showed that lysine residues located at the N-terminus did not significantly influence the turnover of the protein. Indeed, compared to the relative counterpart NSsΔN (Figure 2B) and NSs-6KR (Figure 6), the new NSs proteins did not exhibit a remarkable increase (*p* > 0.05) of protein accumulation after exposure to CHX (Figure 7A), suggesting that those sites were not critical in determining the NSs protein fate.

As expected, the chase experiments conducted in the presence of the proteasome inhibitor MG-132 and CHX evidenced that the NSs-NKR responded to inhibitor (*p* = 0.008), as lysines in the core region were maintained. However, even the NSs-6KR-NKR variant responded to the drug (*p* = 0.039) (Figure 7B), indicating that some lysine residues targeted for ubiquitination were still present in the protein besides these sequences. Furthermore, supporting the previous data, the inhibitor substantially (*p* = 0.036) augmented NSs-6KR-NKR protein mean half-life (t_1/2_ 19.6 h) compared to the relative mock-treated sample (t_1/2_ 9.8 h). A further investigation by mass-spectrometry revealed the presence of four more lysine residues, located in the C-terminal of the protein, which underwent ubiquitination. In particular, we found ubiquitinated lysines at the 180, 244, 294, and 298 position (Table 1).

## 4. Discussion

The non-structural NSs protein of many *Bunyaviridae* and *Phenuiviridae* family members has been shown to be an important virulence factor, able to counteract host innate immunity to viral infections [13,14,15,16,17,18,19,20,21,57,58].

An important factor affecting protein behavior resides in its stability and turnover. Indeed, a tight control of cell proteins’ half-life is required in order to support an efficient cellular homeostasis, death/survival, and replication. Most of the viral proteins exert their activity by interfering in critical cellular processes, such as signal transduction and cell-cycle progression. Thus, their function is tightly controlled through several viral and cellular mechanisms.

One aspect that determines protein fate is the presence of unfolded domains, which confer a reduced stability and a rapid degradation via lysosome or proteasome systems [68,69,70]. The intrinsically disordered region (IDR) with an unfolded structure described for Bunyamwera virus (BUNV) NSs was associated with the reduced viral protein stability in infected cells [62,63].

Previously, we demonstrated that the C-terminus of TOSV NSs could retain an IDR with a negative effect on protein turnover [58]. In the present work, a deeper investigation of TOSV NSs protein properties was pursued. New data suggest that TOSV NSs shares similarities with BUNV NSs, since its stability is strongly influenced by IDR located at the N-terminus (NSsΔN), along with that at the C-terminal (NSsΔC) part of the protein. The increased stability of NSsΔN and NSsΔC, demonstrated by semi-quantitative immunoblotting, suggested that these domains are important in order to maintain protein stability. Indeed, the cycloheximide (CHX) chase assay allowed us to confirm that external NSs IDRs are strikingly related to protein half-life, and NSsΔN exhibited a longer half-life. Therefore, the unfolded NSs N-terminus drastically compromised protein stability, along with the C-terminus, which, in turn, had a minor effect.

Nevertheless, apart from protein IDRs, other mechanisms involved in protein stability were proposed. Based on Bunyamwera virus (BUNV) studies where the involvement of the ubiquitin-proteasome system in controlling NSs fate was described [62], post-translational modifications (PTMs) in TOSV NSs were considered. Contrary to RVFV and BUNV, TOSV NSs presented many lysine residues that could undergo ubiquitination.

Under specific experimental conditions, we provided direct evidence of TOSV NSs ubiquitination both by immunoblotting and mass spectrometry (MS). We identified the ubiquitin linkage at lysine residue 104, 109, and 154, as they were modified by the signature peptide ‘Gly-Gly’ derived from ubiquitin by MS. Surprisingly, identified PTM sites were located in the core region of the protein, suggesting an incisive role of this region in the protein fate.

However, TOSV NSs ubiquitination on other sites of the protein could be neither demonstrated nor excluded. Despite being more stable, the NSs mutant, lacking all the core lysine residues (NSs-6KR), was still susceptible to MG-132 treatment. This suggested the presence of additional lysine residues, located outside the core region, subjected to ubiquitination. In an attempt to better characterize the NSs post-translational modification by ubiquitination, a deeper investigation was conducted. The mutation of lysine residues located at the amino-terminal part of the protein (NSs-NKR) excluded the presence of ubiquitination targets in this region, since the mutation did not affect the protein degradation rate or half-life, as demonstrated by the CHX chase (Figure 7A).

Further experiments using the NSs-6KR-NKR, lacking all the lysines at the N-terminus and core regions, evidenced the presence of additional ubiquitination sites in the C-terminal part. The CHX chase in the presence of the proteasome inhibitor MG-132 revealed that the NSs-6KR-NKR mutant still responded to the inhibitor, thus ubiquitination still occurred on the protein. More comprehensive analysis by MS identified the ubiquitin-derived ‘Gly-Gly’ signature peptide linked at lysine residues 180, 244, 292, and 298, as they were modified by ubiquitin.

**Figure 8:**
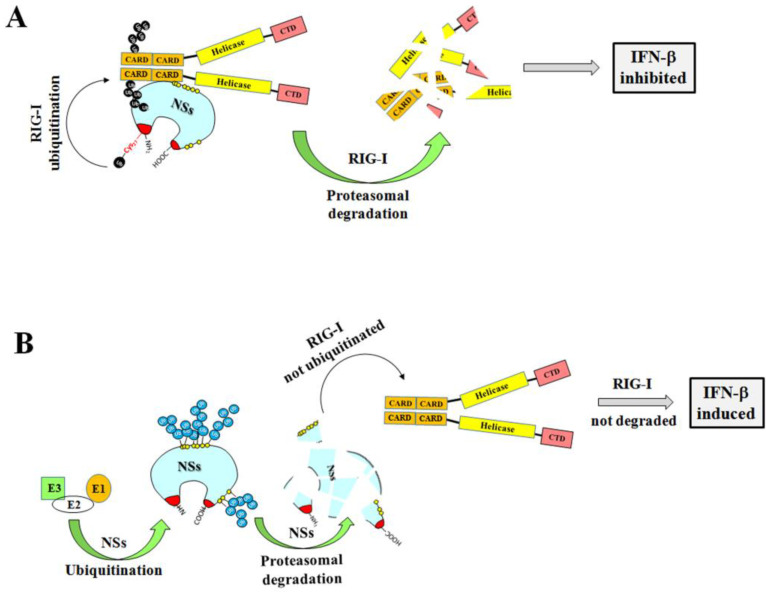
Cartoon representation of the putative function of the TOSV NSs on RIG-I-mediated interferon beta production and the influence of ubiquitination on the viral protein function. (**A**) The TOSV NSs protein accumulated in the cell cytoplasm efficiently interacts with RIG-I and mediates its ubiquitination and subsequent degradation, counteracting IFN-β production. On the contrary (**B**), the high ubiquitination rate of the NSs negatively affects its stability, targeting the protein for proteasomal degradation. Consequently, RIG-I is not depleted and the physiological secretion of IFN-β occurs.

These data open the way to new cellular mechanisms responsible for TOSV NSs functions and to its role in TOSV pathogenicity. Indeed, during the first phases of viral replication, the rapid degradation of the NSs protein by the proteasome could be a regulatory mechanism of cell defense, thus inhibiting RIG-I degradation and allowing the expression of IFN-β in the infected host (Figure 8).

## Figures and Tables

**Table 1:** The enriched NSs protein was analyzed by mass spectrometry in an attempt to identify lysine residues targeted for specific ubiquitination. MS2 mass spectrometry results (TLS, Siena, Italy) are reported, showing the identification of TOSV NSs peptide containing the ubiquitinated lysine residues at position 180, 244, 294, and 298.

Lysine Position	Percentage Sequence Coverage	Peptide Sequence	Peptide Identification Probability	Mascot Ion Score	Mascot Identity Score	Mascot Delta Ion Score	Variable Modifications Identified by Spectrum
**180**	7.21%	VLIEGK***k***HGLTAFDLPGNDILGDICVVQAAR	99.70%	58.6	60.3	36.67597403	K7:GlyGly (+114.04)
**180**	7.21%	VLIEGK***k***HGLTAFDLPGNDILGDICVVQAAR	99.70%	45.9	33.3	32.14675325	K7:GlyGly (+114.04)
**180**	6.94%	***k***HGLTAFDLPGNDILGDICVVQAAR	99.70%	63.2	48.6	55.33246753	K1:GlyGly (+114.04)
**244**	3.80%	KED***k***	99.70%	55.4	60	21.31764706	K4:GlyGly (+114.04)
**244**	6.33%	KED***k***RAKAKGLmSmCAAR	99.70%	51.2	43.7	31.05	K4:GlyGly (+114.04)
**244**	6.33%	ED***k***RAKAKGLMSMCAAR	99.70%	48.7	50.4	30.65454545	K3:GlyGly (+114.04)
**294–298**	6.59%	TDLGFRETALSTFWAKDWPTPQETILSD***k***RcL***k***EDMR	99.70%	48.6	36	34.75324675	K29:GlyGly (+114.04) K33:GlyGly (+114.04)
**294–298**	6.33%	DWPTLQETILSD***k***RcL***k***EDmRVTK	99.70%	52.6	38	43.26406926	K13:GlyGly (+114.04) K17:GlyGly (+114.04)
**294**	6.33%	ETALSTFWAKDWPTPQETILSD***k***	99.70%	60.9	65.5	23.27176471	K23:GlyGly (+114.04)
**298**	6.33%	CL***k***EDMRVTKWLPSPPHYPPL	99.70%	45.8	38.3	27.21315789	K4:GlyGly (+114.04)
**298**	6.33%	CL*K*EDMRVTKWLPSPPHYPPL	99.70%	44.2	45.9	27.91753247	K23:GlyGly (+114.04)
